# The State of the Evidence for Whole-System, Multi-Modality Naturopathic Medicine: A Systematic Scoping Review

**DOI:** 10.1089/acm.2018.0340

**Published:** 2019-02-15

**Authors:** Stephen P. Myers, Vanessa Vigar

**Affiliations:** ^1^ National Centre for Naturopathic Medicine, Southern Cross University, Lismore, Australia.; ^2^ NatMed Research Unit, Division of Research, Southern Cross University, Lismore, Australia.; ^3^ Foundations of Naturopathic Medicine Institute, Snoqualmie, Washington.; ^4^ Integria Healthcare, Ballina, Australia.

**Keywords:** naturopathic medicine, naturopathy, global, systematic review, whole-system, pragmatic

## Abstract

***Objective:*** To summarize the current state of the research evidence for whole-system, multi-modality naturopathic medicine.

***Design:*** A systematic search for research articles from around the world was undertaken using MEDLINE, Embase, CINAHL, AMED, and WHO regional indexes. Naturopathic journals and gray literature were hand searched. No language restrictions were imposed.

***Interventions:*** All human research evaluating the effectiveness of naturopathic medicine, where two or more naturopathic modalities are delivered by naturopathic clinicians, were included in the review. Case studies of five or more cases were included.

***Results:*** Thirty-three published studies (*n* = 9859) met inclusion criteria (11 American; 4 Canadian; 6 German; 7 Indian; 3 Australian; 1 United Kingdom; and 1 Japanese) across a range of mainly chronic clinical conditions. The studies predominantly showed evidence for the efficacy of naturopathic medicine for the conditions and settings in which they were based.

***Conclusions:*** To date, research in whole-system, multi-modality naturopathic medicine shows that it is effective for treating cardiovascular disease, musculoskeletal pain, type 2 diabetes, polycystic ovary syndrome, depression, anxiety, and a range of complex chronic conditions.

## Introduction

Naturopathic medicine is an eclectic practice of health care united by core underlying philosophy, theory, and principles. A central tenet of naturopathic philosophy is *vis medicatrix naturae* (the healing power of nature), an ancient concept often ascribed to Hippocrates,^1,2^ that refers to an inherent, self-organizing healing process in living systems which establishes, maintains, and restores health.^1^

The terms “whole-system” & “multi-modality” within the context of naturopathic medicine are outlined in Table 1. Broadly these refer to the practice of naturopathic medicine as a complex health care intervention, which utilizes a combination of clinical modalities (or therapies) in the treatment of each individual. This contrasts with a single modality approach where only one therapy is used. Modalities used in naturopathic practice are determined by a structured system of theory and principles based within its philosophy; refer to Table 1 for a list of core modalities.

**Table 1.** Definitions for Key Study Components

Naturopathic medicine	The WNF defines the naturopathic profession based on two fundamental philosophies of medicine (vitalism and holism) and seven principles of practice (healing power of nature; treat the whole person; treat the cause; first, do no harm; doctor as teacher; health promotion and disease prevention; and wellness).^10^ The philosophy, theory, and principles are translated to clinical practice through a range of therapeutic modalities. The WNF has identified seven core modalities: (1) clinical nutrition and diet modification/counseling; (2) applied nutrition (use of dietary supplements, traditional medicines, and natural health care products); (3) herbal medicine; (4) lifestyle counseling; (5) hydrotherapy; (6) homeopathy, including complex homeopathy; and (7) physical modalities (based on the treatment modalities taught and allowed in each jurisdiction, including yoga, naturopathic manipulation, and muscle release techniques).^10^ This scoping study is limited to naturopathic medicine as defined and encompassed by the WNF. Other systems of traditional medicine, such as Traditional Chinese Medicine and Ayurveda, are not included in this systematic scoping review study.
Multi-modality	Within the context of this systematic scoping review, “multi-modality naturopathic practice” was defined as including a minimum of two modalities as part of a single clinical approach to treatment of an individual. The practice of a single modality was considered to be more indicative of that specific modality, rather than eclectic naturopathic general practice.
Whole system	Refers to the practice of naturopathic medicine as a complex health care intervention that addresses simultaneously the multiple dimensions (physical, mental, spiritual, family, community, and environment) of an individual patient^1^ as pragmatically practiced by naturopathic clinicians.

WNF, World Naturopathic Federation.

The World Health Organization (WHO) defines naturopathy as part of Traditional and Complementary Medicine (T&CM) and has recommended this sector to build evidence to support its safe and effective use.^3^ The imperative to increase the evidence base of T&CM results from the emergence of evidence-based medicine (EBM) in the last quarter of the twentieth century.^4^ While a substantial body of evidence for the effectiveness of the “tools of trade” of naturopathic medicine (i.e., herbal and nutritional supplements; and lifestyle interventions [LIs]) is now available, there exists little quantitative scientific evidence documenting it as an effective medical practice.^5^

The movement toward developing a scientific evidence base for naturopathic medicine is not without controversy. Some have argued that EBM is antithetical to naturopathy, out of concern that traditional naturopathic philosophy and practice will be marginalized or excluded in a process of coercing nonorthodox systems of health and healing to fit into the mainstream scientific paradigm.^6^ Others argue that although there exist inevitable tensions between T&CM and EBM epistemologies, these tensions and their resolutions also can hold the key to a more productive understanding between traditional and scientific knowledge.^7^

Galvanized by this need to develop a body of quantitative scientific evidence supporting naturopathic medicine, a group of U.S. naturopathic researchers received a grant from the National Institutes of Health's National Center for Complementary and Integrative Health (previously the National Center for Complementary and Alternative Medicine) in 2006 to develop a Naturopathic Medical Research Agenda (NMRA).^8^ The project involved research directors from every North American institution with a naturopathic program, Southern Cross University (an Australian institution with a publicly funded naturopathic medicine program), along with 1200 naturopathic academics, practitioners, students, and selected medical researchers. The primary recommendation from the NMRA was that research be conducted on the whole practice of naturopathic medicine, rather than on single agents (such as individual herbal or nutritional supplements).^8^

Following the NMRA recommendations, significant research occurred, especially in North America. In 2015, a systematic review of this research,^9^ including 15 clinical studies reporting on the outcomes of multi-modality treatment delivered by North American naturopathic physicians, was published. They concluded that while many sample sizes were small, results indicated that receiving whole-system naturopathic medicine was associated with improved health outcomes and improved quality of life (QOL) in patients with or at risk for chronic conditions, including cardiovascular disease (CVD), type 2 diabetes, chronic pain, anxiety, multiple sclerosis, hepatitis C, and menopausal symptoms.^9^ Also in 2015,^9^ the World Naturopathic Federation (WNF) convened its inaugural meeting. The WNF now represents more than 50 international naturopathic organizations with a primary goal to promote and advance the naturopathic profession. Given this international interest, it is timely to undertake a systematic scoping review that summarizes the state of the evidence for whole-system, multi-modality naturopathic medicine across the world. A systematic scoping review differs from a systematic review in that it sets out to examine the extent, range, and nature of research activity in a broad area,^10^ while a systematic review generally sets out to answer a focused question by synthesizing all available research. The main goal of this scoping study is to highlight the breadth of the quantitative scientific research in naturopathic medicine.

## Methods

**FIG. 1.**
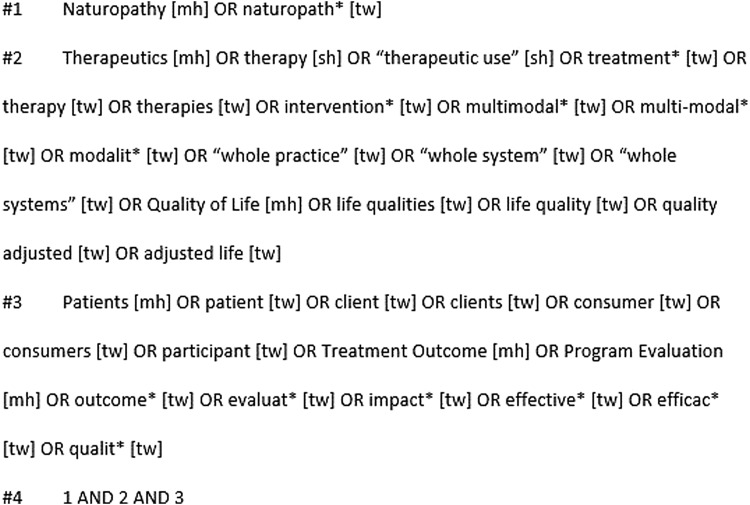
Medline Search Strategy.

In July 2018, the authors undertook a comprehensive search of MEDLINE, Embase, CINAHL, AMED, and the WHO regional indexes (AIM, LILACS, IMEMR, IMSEAR, WPRIM). The MEDLINE search strategy is shown in Figure 1; other search strings are available upon request.

In addition, the authors conducted additional hand searches of the following journals: *British Naturopathic Journal*, *Townsend Letters*, *Journal of the Australian Traditional Medicine Society*, *The International Journal of Naturopathic Medicine*, and the *Journal of Orthomolecular Medicine*. Submissions to the Australian *Natural Therapy Review* regarding the effectiveness of naturopathic medicine^11^ were also searched for additional references.

Studies were included if they met the following criteria:

(1) Controlled clinical trials, longitudinal cohort studies, observational trials, or case series involving five or more cases presented in any language
(2) Human studies
(3) Multi-modality treatment administered by a naturopath (naturopathic clinician, naturopathic physician) as an intervention
(4) Non-English language studies in which an English title and abstract provided sufficient information to determine effectiveness
(5) Case series in which five or more individual cases were pooled and authors provided a summative discussion of the cases in the context of naturopathic medicine

It was decided that case series with less than five cases were more representative of the individual cases, rather than an evaluation of whole-system naturopathic treatment in a specific condition.

Titles and abstracts were screened by both authors, based on the inclusion criteria, with disagreements settled by discussion.

**FIG. 2.**
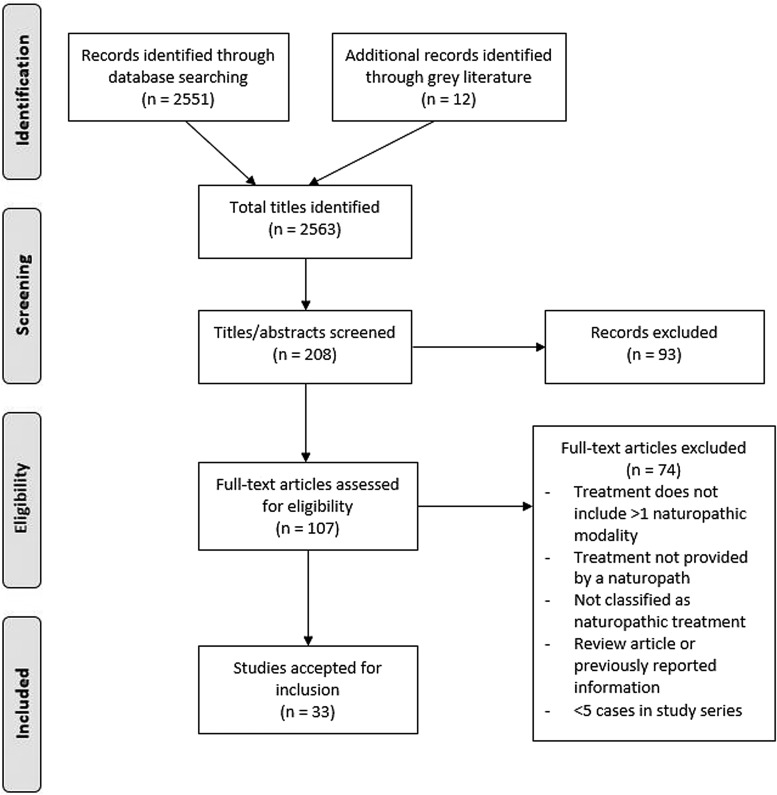
Flow diagram of study selection.

See Figure 2 for a flowchart documenting the study selection.

### Data extraction

The following data from included studies were extracted (V.V.) and summarized using a data extraction sheet: study reference, origin, condition, number of participants, study design, primary outcomes, patient-reported outcomes, and modalities utilized. Data were divided by conditions treated (Tables 2–8).

**Table 2.** Cardiovascular Disease

*Author/Year*	*Country*	*Condition*	N	*Design (and comparator)*	*Outcomes*	*Results*	*Modalities*
Edla 2016	India	Hypertension	104	Prospective cohort study. Hypertensive patients enrolled into a yoga and integrated naturopathy hospital over a 3-month period. All had 15 days in care and 3-month follow-up.	Blood pressure, anthropometry, cholesterol, Tgs, and HbA1C. Primary study outcome—BP control plus 50% or greater reduction in dose of antihypertensive medication relative to baseline. Compliance.	79 (99%) achieved target BP control (<140/90 mmHg) and 66 (83%) achieved this with >50% reduction in antihypertensive medication dose, and in 8 (10%) patients, all medications were withdrawn. Reduction in triglycerides was significant mean difference of −19.3 (85.4) (*p* < 0.001), and HbA1C was significantly reduced among diabetics.	A structured residential package of vegetarian diet, yoga-based exercise, and patient education in addition to pharmacotherapy.
Braun 2014	Australia	Cardiac surgery patients—postoperative outcomes	337	Prospective cohort study—Integrative cardiac wellness program (ICWP) (*n* = 337), delivered to patients undergoing cardiac surgery, compared with matched cohort receiving ‘usual care’ from previous years (*n* = 585).	Postsurgery outcomes: 24 hr serum troponin I, inotrope requirements, low output state, atrial fibrillation (AF), hospital length of stay (LOS), blood loss.	ICWP had significantly reduced requirement for postsurgery inotropic support (*p* < 0.0001), a reduced incidence of low output state (*p* = 0.025) and a reduced rate of atrial fibrillation (*p* = 0.029) in comparison to ‘usual care’ group. After adjusting for baseline imbalances by multivariate analysis, the reduction in inotropic support remained significant (*p* < 0.001) with a relative reduction of 42%.	Standardized nutritional supplement regime with short individualized naturopathic consultation (10–20 min) to discuss diet and lifestyle change.
Seely 2013	Canada	CVD	246	Randomized controlled trial—2 arms: - usual care (UC) - adjunct naturopathic care (NC-enhanced usual care) Prevention of CVD in cohort at increased risk of CVD—1 year evaluation of risk profile	Framingham risk score, prevalent metabolic syndrome, SF-36	Compared with participants in the UC group, at 52 weeks those in the NC group had a reduced adjusted 10-year cardiovascular risk UC: 10.81%; NC: 7.74%; risk reduction–3.07% [95% confidence interval (CI): −4.35% to −1.78%], *p* < 0.001, and a lower adjusted frequency of metabolic syndrome UC: 48.48%; NC: 31.58%; risk reduction–16.90% [95% CI: −29.55% to −4.25%], *p* = 0.002.	Individualized treatment, including specific diet and lifestyle recommendations, and the prescription of selected natural health products
Nandakumar 2012	India	CVD risk factors	72	POSTER PRESENTATION. Pilot study of 3-week yoga and naturopathy intervention in patients with known CVD risk factors. Active group compared to wait-listed control.	BP, BMI, lipid profile, blood glucose, and psychologic measures such as Hospital Anxiety and Depression Scale (HADS). Somatization component of SCL90 and general health perception	Significant reductions (*p* < 0.05) in systolic blood pressure (140.36 vs. 124.62), diastolic blood pressure (85.28 vs.76.93), fasting blood glucose (142.29 vs. 116.61), postprandial blood glucose (233.2 vs. 172.19), body mass index (33.05 vs. 31.86), total cholesterol (181.61 vs. 161.04), LDL cholesterol (107.76 vs. 85.72),triglycerides (152.8 vs. 131.74), anxiety (6.79 vs. 4.98), depression (6.54 vs. 4.45), and somatization symptoms (7.84 vs. 3.56) compared to wait-listed control at 3 weeks.	Yoga primary treatment. Naturopathy based lifestyle intervention.
Murthy 2011	India	Hypertension	104	Prospective cohort study. Inpatient treatment with naturopathy and yoga for 3 weeks in patients with mild-to-moderate hypertension. 1-year follow-up. Medication was removed where possible.	Blood pressure, body weight, lipid profile	Mean systolic blood pressure on admission was 139.6 ± 16.2, which reduced to 129.6 ± 10.4 on discharge, diastolic reduced from 91.2 ± 9.8 to 86.1 ± 5.6, (*p* < 0.001). Of these (93% achieved ‘normal’ blood pressure of which 54% were no longer receiving antihypertensive medication). All patients were followed up for a period of 1 year postdischarge. The number of patients who reported for follow-up were 74 (71.1%) at 3 months, 68 (65%) at 6 months, 62 (57%) at 9 months, and 57 (54%) at 1 year. The percentage of patients maintaining normal blood pressure without medication decreased over the 12-month period to 24% at 12 months (of those that returned for follow-up).	Residential yoga and naturopathy based lifestyle intervention.
Bradley 2011	United States	Hypertension	85	Retrospective cohort study. Patients with stage 1 and stage 2 hypertension, with concurrent use of medication. Minimum 6 months of naturopathic care	Blood pressure - systolic (SBP) & diastolic (DBP)	Patients with both stage 1 and stage 2 hypertension appeared to improve during care, with stage 2 patients achieving mean reductions of −26 mmHg (*p* < 0.0001) and −11 mmHg (*p* < 0.0001) in SBP and DBP, respectively. The proportion of patients achieving control (<140/90 mmHg) in both SBP and DBP was increased significantly from 14% to 44% (*p* < 0.033).	Diet, exercise, and preventive counseling were commonly prescribed. Nutritional and botanical supplementation was also commonly prescribed.

CVD, cardiovascular disease.

**Table 3.** Type 2 Diabetes Mellitus

*Author/Year*	*Country*	*Condition*	N	*Design (and comparator)*	*Outcomes*	*Results*	*Modalities*
Bairy 2016	India	T2DM	101	Prospective cohort study. Diabetics admitted to yoga/naturopathic hospital for treatment (15- or 30-day stay). 3 month follow-up.	Achievement of glycemic control along with atleast 50% reduction in dose of antidiabetes medication at 3 months relative to line	Glycemic control parameters measured at baseline and at 3 months, showed significant mean reductions in levels of HbA1C, FBS, and PPBS, respectively, 0.9%, 39 mg/dL, and 36 mg/dL (*p* < 0.001). This effect was more pronounced among patients with a baseline HbA1c of ≥7%, with mean reductions of 1.2%, 53 mg/dL, and 53 mg/dL for HbA1c, FBS, and PPBS, respectively. There was a dose–response relationship between compliance to dietary practices and HbA1c at 3 months with mean reductions of 0.4%, 1.1%, and 1.7% among those with poor, moderate, and excellent compliance to dietary practices, respectively.	A structured residential package of vegetarian diet, yoga-based exercise, and patient education in addition to pharmacotherapy.
Bradley 2012	United States	Diabetes	40	Prospective cohort study for adjunctive care with naturopathic medicine (ANC, *n* = 40) vs. controls (Usual care UC, *n* = 329), electronically matched from electronic health records) — 1 year evaluation of effect on diabetes parameters in patients with inadequately controlled diabetes.	HbA1C, Diabetes self-care assessment scale (SDSCA), PHQ-8 depression scale	Improvements were noted in ANC group in self-monitoring of glucose, diet, self-efficacy, motivation, and mood in SDSCA (*p* = 0.001). Mean HbA1c decreased by −0.90% (*p* = 0.02) in the ANC cohort at 6 months, a −0.51% mean difference compared to usual care (*p* = 0.07). Reductions at 12 months were not statistically significant (−0.34% in the ANC cohort, *p* = 0.14; −0.37% difference compared to the usual care cohort, *p* = 0.12). Medication prescription and utilization for diabetes also increased in the ANC group, which may have contributed to better management of glucose levels.	NDs were instructed to deliver their typical care to participants. Diet and lifestyle advice prescribed in nearly all cases, nutritional supplementation in 74% of patients, and other individualized care plans. Pharmacotherapy was adjusted (increased, decreased, or changed) as needed.
Bradley 2009	United States	Diabetes	37	Retrospective cohort study. Type 2 diabetic (T2DM) patients, with mean duration of care 27 months and average of 11 visits. Care for T2DM was predominantly adjunctive care (80%) versus primary care (20%) during observation period.	HbA1C, lipids, blood pressure	Significant mean changes were: −0.65% for HbA1c (*p* = 0.046), −45 mg/dL for TAG (*p* = 0.037), −7 mmHg in SBP (*p* = 0.02), and −5 mm Hg in DBP (*p* = 0.003) from baseline to last visit. Cholesterol levels showed no significant difference.	Diet, lifestyle, stress management, and nutritional and herbal supplementation were commonly prescribed. Pharmacotherapy was adjusted (increased, decreased, or changed) as needed.
Bradley 2006	United States	Diabetes	16	Retrospective cohort study. T2DM patients with at least 6 months of naturopathic treatment evaluated.	% HbA1C, % of patients reaching adequate glycemic control, or improvement in glycemic control.	31% achieved HbA1C <7%, 61% achieved 7–10%, and 8% were >10%. There were positive controls of cholesterol and triglyceride measures, as well as 44% achieving ‘good control’ of blood pressure. 44% of the population was also taking oral antidiabetic medication and 40% taking insulin.	100% received dietary counseling, 69% were taught stress reduction techniques, and 94% were prescribed exercise. Patients additionally received prescriptions for botanical and nutritional supplementation, often in combination with conventional medication.

**Table 4.** Musculoskeletal Pain

*Author/Year*	*Country*	*Condition*	N	*Design (and comparator)*	*Outcomes*	*Results*	*Modalities*
Stange 2012	Germany	Musculoskeletal pain	221 (ITT) 101 (pp)	POSTER PRESENTATION Prospective cohort study. Patients with chronic musculoskeletal pain (duration >2 years), treated for 2 weeks approximately as inpatient in integrative hospital. Outcome measured at admittance to hospital and 1 year post inpatient treatment. Treatment included allopathic medicine, with integrative CAM treatment.	Pain as measured on a visual analog scale (VAS). QOL measured with SF-36	Mean pain VAS decreased by 15.1 from 60.7 ± 23.0 (admission) to 45.6 ± 26.2 (1 year post-treatment) (*p* < 0.0001, two-sided *t* test), with highest improvement for low back pain (decrease of 17.5) and no differentiation for multi-morbidity (*n* = 46 with, *n* = 55 without). SF-36 physical and mental component scores improved significantly from 40.0 ± 12.2 to 44.3 ± 12.5 and from 29.6 ± 8.2 to 32.9 ± 10.5, respectively (*p* < 0.0001 for each).	Diet and fasting, physical therapy, relaxation, herbs, acupuncture, and neural therapy.
Wiebelitz 2011	Germany	Chronic back pain	161	Prospective cohort study. Naturopathic (*n* = 161) vs. orthopedic (*n* = 187) care of back pain. Outcome measured at discharge, 3 months, and 6 months.	Oswestry score and SF-36. Number of specialist consultations, medication, psychotherapy costs	For the main and secondary objectives, there was no significant difference between groups. Oswestry Score at T0: 43.0, 95% CI: 39.6–45.3; at T2: 40.7, 95% CI: 37.4–44.0). Only in the subgroup of women, who account for 86% of the naturopathic patients, a difference in the main objective (Oswestry Score) was found (*p* = 0.014) in favor of naturopathy (T2: 32.6, 95% CI: 24.9–40.2) compared to orthopedics (T2: 45.1, 95% CI: 41.2–49.1). Treatment results of naturopathic inpatient treatment of chronic back pain are comparable to conventional orthopedic treatment at all points of time.	Diet, physical therapy, lifestyle advice, psychotherapy, and phytotherapy.
Szczurko 2009	Canada	Rotator cuff tendinitis	85	Randomized controlled trial—2 arms: - standardized physical exercises with placebo supplementation (PC) - naturopathic care (NC) Canadian postal workers with rotator cuff tendinitis treated for 12 weeks.	Shoulder Pain and Disability Index (SPADI), SF-36	Final total SPADI scores decreased by 54.5% (*p* < 0.0001) in the NC group and by 18% (*p* = 0.0241) in the PC group. The SF-36 showed statistically significant differences (*p* < 0.01) between the NC and PC groups in all subcategories except social functioning, which showed a trend toward improvement (*p* = 0.038). The NC group showed the greatest improvement over the PC group in role physical (*p* = 0.0015), bodily pain (*p* = 0.0004), and in role emotional (*p* = 0.0020).	Dietary counseling, acupuncture, and Phlogenzym (2 tablets thrice/day)—not an ‘individualized ‘naturopathic’ model of treatment.
Shinto 2008	United States	Multiple sclerosis	45	Randomized controlled trial—6 months, 3 arms: - naturopathic medicine (nm) - usual care (UC) - UC + education	Modified fatigue impact scale, Beck depression inventory (BDI), cognition tests, neurologic impairment (EDSS), multiple sclerosis functionality, SF-36	No significant differences between groups on any outcome measure. A trend favoring the naturopathic care group in the general health subscale of the SF-36 (*p* = 0.11), timed walk (*p* = 0.11), and neurologic impairment (*p* = 0.07).	Set naturopathic intervention: multivitamin/mineral without iron, vitamin C, vitamin E, fish oil, and α-lipoic acid and intramuscular vitamin B12 once a week. Specific diet.
Ritenbaugh 2008	United States	Temporomandibular disorders (TMDs)	160	Randomized controlled trial—3 arms: - usual care (UC) - Traditional Chinese Medicine (TCM) - naturopathic medicine (NM) TCM-20 treatments NM-10 appointments	Worst/average score of facial pain, interference with activities, effect of pain on Activities of Daily Life (ADL)	Overall, reductions in worst pain at end of treatment and 3 months post-treatment were 13% and 22% for UC, 29% and 33% for TCM, and 28% and 39% for NM. Overall, the average pain reductions at end of treatment and 3 months post-treatment were, respectively, 16% and 27% in SC, 35% and 42% in TCM, and 28% and 40% in NM. No significant difference in any of the treatment arms for changes in ADL.	TCM (acupuncture, herbs, tuina, relaxation). NM (herbs, nutrients, and physical medicine). All treatments were individualized.
Szczurko 2007	Canada	Chronic low back pain	75	Randomized controlled trial—2 arms: - standardized physiotherapy program (PC - education and instruction on physiotherapy exercises) - naturopathic care (NC) Canadian postal workers with lower back pain >6 weeks, treatment for 12 weeks.	Oswestry Low Back Pain Disability Questionnaire (OLBPD), SF-36	NC group reported significantly lower back pain (26.89, 95% CI: 29.23–23.54, *p* = 0.0001) than the physiotherapy group. The difference in mean change scores in the OLBPD from baseline to week 12 resulted in a significant reduction of disability in the naturopathic care group compared to the control group (median change = 25; *p* = 0.0001). QOL was also significantly improved in the group receiving NC in all domains except for vitality.	Dietary counseling, deep breathing relaxation techniques, and acupuncture.
Secor 2004	United States	Pain from any cause, as a feature of admission	94	Prospective cohort study—3 arms: - acupuncture (AC) - chiropractic (CC) - naturopathic medicine (NM) All patients had ≥3 treatments and a starting visual analog score (VAS) pain level of >2.	Pain VAS, SF-12	From baseline to end of study, the VAS average across all arms of the study was significantly reduced by 49% from 4.4 to 2.3 (*p* < 0.0001). Acupuncture reduced from 5.0 to 3.3 (34%); Chiropractic from 4.2 to 2.0 (52%); naturopathic 60% pain reduction (*p* < 0.0001). SF-12 — significant improvement overall in physical health seen in all modalities (*p* < 0.0001) and for the individual modalities of acupuncture (*p* < 0.009) and chiropractic (*p* < 0.005). Mental health had no significant change for all treatments.	Acupuncture, chiropractic, or naturopathic treatment. Naturopathic treatment included nutritional supplementation, herbal medicine, nutrition, exercise, physical medicine modalities, possibly prescription drugs.

QOL, quality of life.

**Table 5.** Mood Disorders

*Author/Year*	*Country*	*Condition*	N	*Design (and comparator)*	*Outcomes*	*Results*	*Modalities*
Breed 2017	United States	Depression and Anxiety	60	Prospective cohort study. Patients attending community health center for anxiety or depression treatment. Those that attended >2 visits had data analyzed, mean number of visits = 3.3. During the study period, all 60 participants saw a naturopathic physician (25% saw only a naturopath), and 55% also saw a conventional provider.	Patient Health Questionnaire (PHQ-9) for depression. Generalized Anxiety Disorder 7-item scale (GAD-7) for anxiety. Response rate measured by those with a 50% decrease in score from an initial score of ≥10	Mean baseline PHQ-9 was 16.4 (i.e., moderate-to-severe depression) Mean baseline GAD-7 score was 12.4 (i.e., moderate anxiety). Improvement of symptoms over time (16.4 vs. 8.6, *p* < 0.0001) for depression and for anxiety (12.4 vs. 7.2, *p* < 0.0001). The average change overall was 48% decrease for depression and 42% decrease for anxiety. The primary outcome of >50% decrease from initial scores was achieved by 58.6% of depression patients and 50% of anxiety patients. The overall improvement in symptoms of depression and anxiety was highly significant (*p* < 0.0001).	Modalities included all types of Nth American naturopathic care. Most commonly used were nutraceuticals (75%), homeopathy (30%), herbals (25%), and acupuncture (20%). Pharmaceutic medication was also prescribed.
Gurevich 2015	United States	Bipolar disorder	7	Case series—7 patients that had poor response or significant side effects from conventional medication. Retrospective analysis of patients with treatment >1 year.	Withdrawal of medication. Mental/mood state. Clinical Global Impression Score for Improvement (CGI-I)	Psychotropic medications were gradually eliminated with naturopathic support. CGI-I scores improved from average of 5 down to 1–2 for all patients. No patients suffered psychotic episodes or hospitalization during treatment period. Long-term maintenance of bipolar disorder.	Phytotherapy, vitamin/mineral supplementation, acupuncture, homeopathy, diet modification, exercise, and regulation of sleep cycles. Guided imagery, energetic/spiritual therapies, and psychotherapy were used as needed.
Sarris 2014	Australia	Anxiety & mood	15	Pilot observational study of naturopathic care in private naturopathic practice. Data from 15 naturopaths over 31 consultations was pooled for analysis, with only 8 return consultations being reported.	DASS-21, POMS-65, General Health Questionnaire (GHQ-28)	A significant effect occurred for time, with patients having a reduction of patient-rated depression, anxiety, and stress on the DASS-21. Specifically, depression was reduced by −9.50 (F = 18.13, *p* = 0.002); anxiety by −9.25 points (F = 13.78, *p* = 0.005), and stress by −12.00 points (F = 18.80, *p* = 0.002). On the POMS-65, total mood disturbance was significantly reduced across Time by −33.13 points (F = 11.66, *p* = 0.001). On the GHQ-28, a significant reduction occurred for patient-rated somatic symptoms −5.75 (F = 5.46, *p* = 0.018), anxiety and insomnia symptoms −6.25 (F = 14.71, *p* < 0.001), and social dysfunction −9.50 (F = 14.02, *p* < 0.001).	Herbal, nutritional, lifestyle advice, diet advice. Individualized treatment plans.
Cooley 2009	Canada	Anxiety	75	Randomized controlled trial—2 arms: - standardized psychotherapy care with placebo supplementation (PT) - naturopathic care (NC) Employees with moderate-to-severe anxiety (>6 weeks). The NC was a set regimen and not individualized to each person.	Beck Anxiety Inventory (BAI), SF-36, Measure Yourself Medical Outcome Profile (MYMOP)	Beck Anxiety Inventory (BAI) decreased by 56.5% (*p* < 0.0001) in the NC group and 30.5% (*p* < 0.0001) in the PT group from baseline to study end, as well as in the subscales of the BAI, showing greater improvement compared to PT. Patient centered outcomes as measured by the MYMOP questionnaire showed significant reductions in symptoms 1 (*p* < 0.0001) and symptom 2 (*p* = 0.012) in the NC group compared to the PT group.	Dietary counseling, deep breathing relaxation techniques, a standard multivitamin, and *Withania somnifera* (600 mg per day of standardized root). Not an ‘individualized ‘naturopathic’ model. The PT intervention received psychotherapy, matched deep breathing relaxation techniques, and placebo tablets.

**Table 6.** Complex Chronic Disease

*Author/Year*	*Country*	*Condition*	N	*Design (and comparator)*	*Outcome*	*Results*	*Modalities*
Teut 2013	Germany	Older adults in nursing homes, mixed etiology	58	Pragmatic pilot study, cluster randomized. Older adults living in nursing home type situation, randomized to usual care (UC) or additional integrative medicine (IM) regimes over 12 months. Average age 76.0 ± 12.8 (UC) and 82.7 ± 8.6 (IM)	Nurses Observation Scale for Geriatric Patients (NOSGER); Assessment of Motor and Process Skills; Barthel Index; Qualidem; and Mini-mental State Examination. Profile of well-being	After 12 months, effect sizes in the IM group in comparison to UC were increased in the following areas. Effect size ≥0.3 were observed for activities of daily living on the NOSGER-Activities of Daily Living subscale (0.53), Barthel Index (0.30), Qualidem total sum score (0.39), Profile of Well-being (0.36), NOSGER-Impaired Social Behavior (0.47), and NOSGER-Depressed Mood subscales (0.40). Smaller or no effects were observed for all other outcomes.	Multifaceted treatment plan. Weekly 60-min exercise; naturopathic care (teas, wraps, compresses, herbal massage); fresh fruit/veg juices; homeopathy. Changes in medications were prescribed by the IM doctors involved. Medical doctors and nurses administered most of the therapy, one naturopath involved.
Weidenhammer 2007	Germany	Rehabilitation clinic—various chronic conditions	5278	Prospective cohort study. Evaluation of naturopathic treatment effect on chronic conditions (all of the patients admitted to the hospital over 2 years) Follow-up discharge, 2mth, 6mth, and 12mth.	Various, SF-36	The intensity of the main complaint decreased from 59 ± 25 by an average of 25 points at discharge (19 points at 6-month follow-up) corresponding to an effect size of 0.86 (0.62 at 6-month follow-up). At 6-month follow-up about half of the patients showed a clinically relevant improvement of quality of life (SF-36 sum scores increased by 5 or more points). Intake of drugs was reduced, the number of days off work had decreased after rehabilitation. Patients' satisfaction was ‘good’ on average; with respect to food satisfaction it was lower.	Phytotherapy, lifestyle, and hydrotherapy—adapted to the individual.
Isbell 2007	UK	Mixed etiology	49	Retrospective evaluation of patient assessed improvement in main symptoms after treatment at a multidisciplinary complementary therapies clinic. Primarily musculoskeletal problems treated. All patients with >2 treatments over 3 years evaluated.	MYMOP evaluation of all patients	Between first and last consultation, 57% (*n* = 28) rated an improvement in their first symptom MYMOP and 35% rated an improvement in their overall well-being score. Before treatment 37% of the sample (*n* = 46) rated their symptoms as 3 or less. After treatment 78% of patients rated their symptoms as 3 or less.	Naturopathy, osteopathy, and CranioSacral therapy clinic—there was no differentiation between the different treatments reported.
Ostermann 2002	Germany	Multiple - rheumatic, metabolic, & allergic-type disease	1026	Prospective cohort study. Blankenstein naturopathic hospital evaluating effectiveness of treatment. >3 week inpatient naturopathic treatment. Outcomes evaluated on admission, discharge, and 3 months and 6 months after discharge.	QOL (HLQ & SF-36), mood, physical complaints (GBB24), and pain perception (SES)	All subscales, as well as the total scores of the psychometric test instruments, showed highly significant changes (t test, *p* < 0.01) between the time ‘hospitalization’ and ‘discharge’. Within the follow-up these values were stabilized on a level significantly higher than the initial level. Pain decreased on average from 26 on admittance to 15 on discharge with a level between 20 and 22 at 3- and 6-month follow-up. HLQ and SF-36 were both higher on discharge than at 3 or 6 months follow-up, but still significantly higher than on admission.	Phytotherapy, lifestyle, and hydrotherapy—adapted to the individual.
Beer 2001	Germany	Acute inpatient naturopathic care	618	Prospective cohort study. Inpatient naturopathic treatment (Blankenstein hospital). Three weeks of treatment with outcomes evaluated on admission, discharge, 3 months, & 6 months.	Quality of life—measured by short form-36 (SF-36) and Herdecke Questionnaire for Quality of Life (HLQ)	HLQ improved from baseline score of 50–58 on discharge and dropped to 55 at 3 months and 54 at 6 months, indicating that the improvement was at least partially sustained. SF-36 showed improvement in some subdomains after discharge, with physical pain, general health perception, and emotional/social aspects improving further between 3–6 months after discharge. All subscales, as well as the total scores of life quality, showed highly significant improvements (*t* test, *p* < 0.01) between the times of ‘hospitalization’ and ‘discharge’. During follow-up these values stabilized on a level significantly higher than the initial level.	Phytotherapy, lifestyle, and hydrotherapy—adapted to the individual.

QOL, quality of life.

**Table 7.** Asthma

*Author/Year*	*Country*	*Condition*	N	*Design (and comparator)*	*Outcomes*	*Results*	*Modalities*
Rao 2014	India	Asthma	134	Retrospective analysis of bronchial asthma patients’ in-patient naturopathy and yoga program for 21 days, with follow-up monitoring for up to 3 years.	Lung function capacity (FVC, PEFR, VC, FEV1, FEV/FEC %, MVV).	There was a significant increase in FVC, FEV1, and PEFR at all time points postadmission to 6 months, *p* < 0.0035. Changes in PEFR were maintained to the 36-month follow-up. There was also significant increase in the MVV mean values; however only from admission till the date of discharge (*p* < 0.0035).	Yoga primary treatment. Naturopathic care included diet, mud pack, enema, steam treatments, hydrotherapy, and massage. Pharmacologic treatments also prescribed.
Sathyaprabha 2001	India	Bronchial asthma	37	Nonrandomized trial. Patients with asthma followed for 21-day observational period (control) before inpatient treatment for 21 days (yoga/naturopathic care).	Lung function capacity (FVC, PEFR, VC, FEV1, FEV/FEC %, MVV). ESR, eosinophil count.	Medication was withdrawn over the first few days in the treatment hospital. Lung function significantly increased across all measured parameters (*p* < 0.001). Eosinophil counts and ESR also reduced significantly over 3 weeks (*p* < 0.01).	Diet included fasting protocol, nature cure—external herbal treatments (steam bathing, hot packs etc.), yoga >1 hour daily.

**Table 8.** Mixed Conditions

*Author/Year*	*Country*	*Condition*	N	*Design (and comparator)*	*Outcomes*	*Results*	*Modalities*
Cramer 2003	United States	Menopause symptoms	239	Retrospective cohort with 'usual care' (UC) controls (UC—these were electronically matched health records). Patient group consisted of 79 naturopathic patients and 160 conventional patients.	% of patients with improvement in symptoms	In multivariate analyses, patients treated with naturopathy were approximately seven times more likely than conventionally treated patients to report improvement for insomnia (odds ratio [OR], 6.77; 95% CI: 1.71–26.63) and decreased energy (OR, 6.55; 95% CI: 0.96–44.74). Naturopathy patients reported improvement for anxiety (OR, 1.27; 95% CI: 0.63–2.56), hot flashes (OR, 1.40; 95% CI: 0.68–2.88), menstrual changes (OR, 0.98; 95% CI: 0.43–2.24), and vaginal dryness (OR, 0.91; 95% CI: 0.21–3.96) about as frequently as patients who were treated conventionally.	ND physicians are primary care providers with a scope of practice that includes nutritional supplementation, herbal medicine, nutrition, exercise, physical medicine modalities, minor surgery, and pharmacotherapy.
Milliman 2000	United States	Hepatitis C	41	Retrospective review of 41 consecutive cases of Hepatitis C. Of these only 14 did not undergo interferon therapy and were treated with naturopathic methods. Naturopathic treatment for >1 month was required for inclusion.	Alanine aminotransferase (ALT)	Baseline ALT level was 127 U/L (range: 47–256). The average ALT values for patients on the protocol for a minimum of 1 month was 92 U/L (range: 25–235; *p* = 0.026, paired *t* test). Of the 14 patients reported, seven showed an ALT reduction of greater than 25%. The other seven patients had no change or a slight increase in ALT.	Set herbal/nutritional protocol with variation available depending on individual presentation
Niwa 2013	Japan	Hepatocellular carcinoma	101	Retrospective analysis of naturopathic care given to patients with hepatocellular carcinoma. Treatments were individualized naturopathic treatments.	Prolonged survival.	Key finding was that those who were treated with 4 or more different agents had a greatly increased median survival time than those treated with <4. Also, *Cordyceps sinensis* had an exclusive benefit with a dramatically improved survival in those that were given this as part of their treatment.	Predominantly herbal combinations, with Cordyceps being an important one, but also sand bath treatment (infra-red), intravenous multivitamin infusions, and lifestyle counseling.
Arentz 2017	Australia	Polycystic Ovarian Syndrome in overweight women	122	Randomized controlled trial—2 arms: - Lifestyle intervention - Lifestyle intervention plus standardized herbal treatment protocol. 3-month treatment period.	Primary outcome was oligomenorrhea/amenorrhea Secondary outcomes included hormone modulation, anthropometrics, QOL, mood, pregnancy, birth outcomes	At 3 months, women in the combination group recorded a reduction in oligomenorrhea of 32.9% (95% CI: 23.3–42.6, *p* < 0.01) compared with controls. Menstrual cycle length was 43 days (95% CI: 21–65, *p* < 0.001) lower for women in the herbal medicine group than for those in the lifestyle only group. The treatment effect size was estimated as large η2/*p* = 0:11. Other significant improvements were found for BMI (*p* < 0.01); insulin (*p* = 0.02) and luteinizing hormone (*p* = 0.04); blood pressure (*p* = 0.01); quality of life (*p* < 0.01); depression, anxiety, and stress (*p* < 0.01); and pregnancy rates (*p* = 0.01).	Personalized lifestyle plans and life-coaching for all participants. Additional poly-herbal formulation for the combined lifestyle and herbal treatment group.
Pradeep 2016	India	HIV positive patients undergoing antiretroviral (ART) treatment	40	Parallel nonrandomized matched case–control trial—2 arms: - ART + lifestyle counseling - ART + naturopathic care 1-month treatment with results evaluated at 6 months. Patients were matched for age, sex, CD4 counts, and number of years of infection.	CD4 counts	Results: After the end of 6 months, the IG showed significant changes CD4 cell count (*p* = 3.96E-05). The CG also showed a significant improvement in CD4 cell counts (*p* = 0.024) but not of the same magnitude as of IG. An independent *t* test between the groups has shown that the IG was more significant (*p* = 0.047).	A wide range of hydrotherapy treatments were given, as well as yoga, counseling, diet, juice therapy, mudpacks, and sun baths. Patients were given treatments as inpatients for 1 month and then followed these interventions at home until 6 months.

QOL, quality of life.

### Methodological assessment and quality rating

**FIG. 3.**
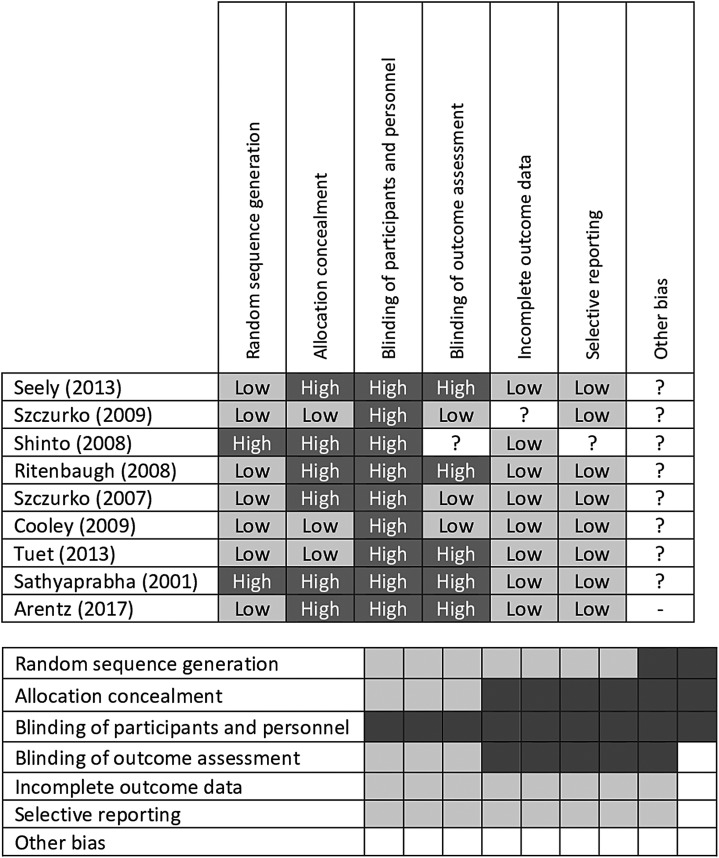
Cochrane risk of bias assessment tables. *gray*, low risk of bias; *light gray*, unclear risk of bias; *dark gray*, high risk of bias

Risk of bias assessment was conducted on all clinical trial reports using the Cochrane Collaboration Risk of Bias tool (Fig. 3).^12^

## Results

### Studies meeting inclusion criteria

A total of 2551 titles were located and the titles and abstracts screened for eligibility. Full text of 95 primary studies and 12 gray literature studies were reviewed in detail (totalling 107). Thirty-three articles were accepted for inclusion, totalling 9859 study participants. The primary reasons for exclusion were lack of multi-modality treatment (i.e., only a single modality was practiced) or the studies did not identify that treatments were conducted by naturopathic clinicians. Notably, studies were excluded because they prescribed naturopathic-style treatments, but were administered by integrative doctors or other personnel not identified as naturopathic. Two poster presentations were included,^13,14^ in which the data were relevant and not reported elsewhere.

### Characteristics of included studies

A majority of the included studies were observational cohort studies (12 prospective and 8 retrospective), with 11 clinical trials and 2 case series (Table 9). Studies were reported from seven different locations: United States (US), Canada, Germany, India, Australia, Japan, and the United Kingdom. The studies are diverse in nature, representing short-term inpatient care (primarily in studies from India and Germany) to longer term observational reports of outpatient treatment (primarily in the US and Canada). The settings of care included private naturopathic practice, integrative hospitals, inpatient health care clinics, or research institutes.

**Table 9.** Clinical Conditions and Study Design Index

*Condition*	*Year*	*Author*	*RCT*	*Noncontrolled trial*	*Prospective cohort*	*Retrospective cohort*	*Case series*
Musculoskeletal pain	2012	Stange				✓	
	2010	Wiebelitz			✓		
	2009	Szczurko	✓				
	2008	Shinto	✓				
	2008	Ritenbaugh	✓				
	2007	Szczurko	✓				
	2004	Secor			✓		
Cardiovascular disease	2016	Edla			✓		
	2014	Braun			✓		
	2013	Seely	✓				
	2012	Nandakumar		✓			
	2011	Bradley				✓	
	2011	Murthy			✓		
Diabetes	2016	Bairy			✓		
	2012	Bradley			✓		
	2009	Bradley				✓	
	2006	Bradley				✓	
Mood	2017	Breed			✓		
	2015	Gurevich					✓
	2014	Sarris			✓		
	2009	Cooley	✓				
Asthma	2014	Rao				✓	
	2001	Sathyaprabha		✓			
Polycystic ovarian syndrome	2017	Arentz	✓				
Cancer	2013	Niwa				✓	
Menopause	2003	Cramer				✓	
Hepatitis C	2000	Milliman					✓
Multiple sclerosis	2008	Shinto	✓				
HIV	2016	Pradeep		✓			
Mixed chronic conditions	2013	Teut	✓				
	2007	Weidenhammer			✓		
	2007	Isbell				✓	
	2002	Ostermann			✓		
	2001	Beer			✓		

RCT, randomized controlled trial.

### Research locations

#### United States

Eleven articles from the US are included in this review. Of these, nine also are included in the Oberg et al. (2015) systematic review of naturopathic whole system practice in North America.^9^ Two additional studies published after the 2015 review also were identified.^15,16^ All studies were of chronic disease conditions: three in chronic pain management of different etiologies^17–19^; three evaluated outcomes in type 2 diabetes management^20–22^; two in mood disorders^15,16^; and one on treatment of hypertension.^23^ The remaining two studies included treatment for hepatitis C^24^ and menopausal symptoms.^25^

Seven publications were prospective or retrospective observational studies,^15,20–25^ three were randomized controlled trials (RCTs),^17–19^ and one was a case series of difficult-to-treat patients.^16^ All the studies were conducted in the community in either public or private naturopathic clinics or in community health centers.

The naturopathic modalities included diet counseling and physical activity recommendations, stress reduction strategies, dietary supplements, hydrotherapy, manual therapy, and botanical medicines.^9^

#### Canada

Four articles were conducted in Canada specifically in a population of Canada Post employees. These included two in chronic pain management,^26,27^ one in anxiety,^28^ and one for primary prevention in CVD.^29^ All of these studies were RCTs, conducted at work-site clinics, and all were included in the Oberg et al. review of 2015. Naturopathic modalities included dietary counseling, nutritional supplementation, and relaxation techniques. One study used acupuncture in addition to the other modalities.^27^

#### Germany

Six articles from Germany were identified for inclusion.^14,30–34^ Two were conducted in the treatment of musculoskeletal pain,^14,33^ three in various chronic conditions of mixed etiology,^31,32,34^ and one examining QOL measures.^35^ Five were conducted in integrative hospitals, and one was conducted in a residential, long-term care setting. The naturopathic care reported in these studies is from integrative inpatient treatment in hospitals where naturopathic methods are used along with conventional medical methods. Four of the studies were observational, while two were RCTs.^31,33^ Naturopathic modalities included dietary interventions (including fasting), botanical medicine, physical therapy, and hydrotherapy as the main interventions, with additional homeopathy,^31^ acupuncture,^14^ and psychotherapy^33^ provided in one study each.

#### India

Seven studies from India^13,36–41^ included three in hypertension or CVD risk,^13,37,38^ two in asthma,^39,40^ one in type 2 diabetes,^36^ and one as an adjuvant to antiretroviral therapy.^41^ All of the Indian studies were inpatient treatments in either naturopathic hospitals or research institutes, with 15–30 days of care. These residential treatments could be classed as highly intensive, with yoga as the primary focus for all of the studies (being practiced up to four sessions per day). Naturopathic modalities included in the treatments varied, but consisted mainly of a specific vegetarian diet (often including a fasting protocol) and topical or inhalation applications that may or may not include botanical medicine. Pharmaceutic medication is also used as needed, with medication being concurrently reduced or withdrawn, as appropriate.

#### Australia

Three studies from Australia were identified.^42–44^ One of these was an observational pilot study of individualized naturopathic care for patients with anxiety and mood disorders conducted across private practice clinics,^42^ with most of the practitioners providing botanical and nutritional medicine along with diet and lifestyle advice. The second study was an open-label prospective cohort study of an integrative cardiac wellness program in patients undergoing cardiac surgery in a hospital setting, which was compared with a matched cohort of patients receiving “usual care”,^43^ and the third was a pragmatic clinical trial in private naturopathic practice.^44^ These latter two studies provided a set naturopathic protocol, including nutritional or botanical medicine in conjunction with personalized lifestyle and dietary planning.

#### United Kingdom

One study was identified from the United Kingdom, which was a retrospective evaluation of patient-assessed improvement in their primary symptom after treatment at a multidisciplinary complementary therapy clinic.^45^ The conditions were primarily musculoskeletal complaints and were treated with naturopathy, osteopathy, or CranioSacral therapy, with no distinction given between the different treatments in the report.

#### Japan

One study was identified from Japan, which was a retrospective analysis of all patients from a particular treatment center with hepatocellular carcinoma. Patients received individualized naturopathic treatment, including the use of medicinal mushrooms and other botanical medicines as the mainstay of treatment, with additional therapies, including intravenous multivitamin infusions and lifestyle counseling.^46^

### Clinical conditions, study design, settings, and modalities

Included studies are categorized by clinical condition and study design (Table 9) and by clinical setting (outpatient and inpatient) and modalities (Table 10).

**Table 10.** Clinical Settings and Modalities Used

					*Modalities prescribed*										
*Country*	*Author/Year*	*Setting*	*Inpatient Y/N*	*Duration (weeks)*	*Pharmaceutic*	*Diet*	*Phytotherapy*	*Nutritional Agents*	*Acupuncture*	*Yoga*	*Hydrotherapy*	*Homeopathy*	*Physical therapy*	*Relaxation exercises*	*Other*
US	Breed 2017	HealthPoint—integrated multidisciplinary community health center	N	Av 3 visits	✓	✓	✓	✓	✓			✓			
	Gurevich 2015	Private psychiatric practice	N	>52		✓	✓	✓	✓			✓			
	Bradley 2012	Group health—integrated health care	N	>52	✓	✓	✓	✓							
	Bradley 2011	Bastyr center for natural health	N	>26		✓	✓	✓							
	Bradley 2009	Bastyr center for natural health	N	∼110	✓	✓	✓	✓							
	Ritenbaugh 2008	Medical clinic	N	16		✓	✓						✓		
	Shinto 2008	Naturopathic clinic	N	26		✓	✓	✓	✓						
	Bradley 2006	Bastyr center for natural health	N	>26	✓	✓	✓	✓							
	Secor 2004	Special care holistic wellness connection-hospital-affiliated outpatient CAM clinic	N	12		✓	✓	✓	✓				✓		
	Cramer 2003	1 CAM Clinic & 6 Conventional Medical Clinics	N	Av 6 visits	✓	✓	✓	✓							
	Milliman 2000	Private naturopathic practice	N	>4		✓	✓								
Canada	Seely 2013	Worksite location — Canada Post employees	N	52		✓	✓	✓	✓						
	Cooley 2009	Worksite location — Canada Post employees	N	12		✓	✓	✓						✓	
	Szczurko 2009	Worksite location — Canada Post employees	N	12		✓	✓	✓						✓	
	Szczurko 2007	Worksite location — Canada Post employees	N	12		✓			✓					✓	
Germany	Teut 2013	Residential care program	Y	52	✓	✓	✓	✓				✓			
	Stange 2012	Centre of Integrative Medicine	Y	52		✓	✓		✓				✓		
	Wiebelitz 2011	Private naturopathic practice	Y	2–3		✓	✓						✓		✓
	Weidenhammer 2007	CAM rehabilitation clinic	Y	Varies		✓	✓				✓				
	Ostermann 2002	Blankenstein hospital	Y	3		✓	✓				✓				
	Beer 2001	Blankenstein hospital	Y	3		✓	✓				✓				
India	Edla 2016	Manthena Satyanarayana Raju Arogyalayam hospital	Y	2	✓	✓				✓					
	Pradeep 2016	HIV sanatorium of National institute of Naturopathy (NIN), Panchagani, Maharashtra	Y	4		✓				✓	✓			✓	✓
	Bairy 2016	Manthena Satyanarayana Raju Arogyalayam hospital	Y	2–4	✓	✓				✓					
	Rao 2014	JindalNatureCure Institute, Bangalore	Y	3	✓	✓				✓	✓				✓
	Nandakumar 2012	Naturopathic clinic	Y	3		✓	✓			✓					
	Murthy 2011	JindalNatureCure Institute, Bangalore	Y	3	✓	✓				✓	✓				✓
	Sathyaprabha 2001	Institute of Naturopathy and Yogic Sciences	Y	3		✓				✓					✓
Australia	Arentz 2017	Private naturopathic practice	N	12		✓	✓						✓	✓	
	Sarris 2014	Private naturopathic practice	N	4–6		✓	✓	✓							
	Braun 2014	The Alfred Hospital	Y	>4	✓	✓		✓							
United Kingdom	Isbell 2007	Multidisciplinary CAM clinic	N	Varies		✓	✓	✓							✓
Japan	Niwa 2013	Hospital records	N	∼58		✓	✓	✓							✓

#### Cardiovascular disease

Six studies were assessed that investigated outcomes for present CVD or development of CVD risk factors, including two RCTs and four prospective cohort studies.

Four of these studies evaluated hypertension as a primary outcome.

- In India, results of two observational inpatient studies on populations with hypertension demonstrated substantive blood pressure control (<140/90 mmHg) after naturopathic treatment.^37,38^ In one study (*n* = 104), 93% of participants achieved control after 21 days (*p* < 0.001),^38^ while a separate study (*n* = 104) found that 99% of participants achieved control after 15 days (*p* < 0.001).^37^ In both studies, results were achieved in addition to simultaneous reduction or elimination of antihypertensive medication in a substantive number of the participants.
- A poster abstract from India reported a randomized waitlisted controlled clinical trial (*n* = 72),^13^ which demonstrated an overall significant reduction (*p* < 0.05) in mean systolic blood pressure (−15 mmHg; 140–125 mmHg) and mean diastolic blood pressure (−8 mmHg; 85–77 mmHg) after 3 weeks of intensive inpatient treatment.^13^
- A US retrospective analysis of outpatients (*n* = 85) treated for hypertension in a naturopathic clinic over 6 months showed a mean reduction of 26 mmHg systolic and 11 mmHg diastolic (*p* < 0.0001) in those with stage 2 hypertension and an overall increase from 14% at baseline to 44% of patients achieving blood pressure control (<140/90 mmHg) over the 6-month treatment period.^23^

Three studies examined multicomponent CVD risk as treatment outcomes.^13,29,43^

- An Australian prospective cohort study of patients undergoing cardiac surgery compared usual care (*n* = 585) against a standardized nutritional supplement treatment combined with individualized diet and lifestyle change (*n* = 337).^43^ Results showed significant postsurgery improvements in cardiac outcomes in the naturopathic care group and a 42% reduction in postsurgery inotropic support (*p* < 0.001).
- A Canadian RCT compared usual care against adjunctive naturopathic care for reducing CVD risk (*n* = 246).^29^ Results showed the 10-year Framingham CVD risk reduced by −3.07%, *p* < 0.001 for the naturopathic over the “usual care” group after 52 weeks. This study also showed a lower frequency of metabolic syndrome (−16.9%, *p* = 0.002) in the naturopathic care cohort compared with usual care.
- In addition to the beneficial effects on blood pressure reported above, the randomized waitlisted controlled clinical trial (*n* = 72)^13^ from India showed reductions in blood glucose, LDL cholesterol, and triglycerides after 3 weeks of residential naturopathic treatment.

Overall, these studies show naturopathic treatment results in a clinically significant benefit for treatment of hypertension, reduction in metabolic syndrome parameters, and improved cardiac outcomes postsurgery.

#### Type 2 diabetes

Four studies on type 2 diabetes mellitus were assessed, including two retrospective and two prospective cohort studies.

In the US, two retrospective studies and one prospective cohort study by the same research group from Bastyr University^20–22^ focused on blood glucose management.

- In the initial retrospective study, all subjects (*n* = 16) received 6 months or more of naturopathic care. Results showed that 31% of patients achieved blood glucose control (HbA1c <7%) and 61% achieved moderate control (HbA1c 7%–10%).^21^
- A second retrospective study (*n* = 37) showed reduced HbA1c of −0.65% (*p* = 0.046), with a mean duration of care of 27 months.^20^ Other significant positive changes were demonstrated for blood pressure (−7 mmHg systolic, *p* = 0.02; and −5 mmHg diastolic, *p* = 0.003) and triglycerides (−45 mg/dL, *p* = 0.037), with no difference in cholesterol.^20^
- A prospective study (*n* = 40) showed reduction in HbA1c of −0.90% (*p* = 0.02) at 6 months after the initial visit.^22^
- One prospective cohort study in India (*n* = 101)^36^ examined inpatient naturopathic treatment for 15–30 days, with patients reviewed again at 3 months. Findings included significant mean reductions of −0.9% in HbA1c (*p* < 0.001) after the initial treatment period and a reduction of −1.7% at 3 months for those with excellent adherence to the treatment provided.

Overall, these studies show naturopathic treatment results in a significant benefit for treatment of diabetes, with reductions in HbA1c that are clinically relevant.

#### Musculoskeletal pain

Six studies of musculoskeletal pain (five articles and one abstract) were included. Clinical conditions included chronic back pain, rotator cuff tendinitis, multiple sclerosis, temporomandibular disorder (TMD), and generalized chronic body pain. Two studies were conducted in Germany,^14,33^ three in the US,^17–19^ and two in Canada.^26,27^

- A Canadian RCT compared 12 weeks of naturopathic care with standard physical therapy (*n* = 75) in employees of Canada Post who had chronic lower back pain. Participants in the naturopathic care cohort showed significant improvement (*p* < 0.0001) in back pain compared with patients receiving standard physical therapy.^26^
- A German controlled prospective cohort study compared naturopathic care with orthopedic care (*n* = 348) in adults who had chronic back pain requiring inpatient treatment. This study showed no differences between naturopathic and standard orthopedic treatment in the whole study population. However, at 3 months, a significant improvement (*p* = < 0.014) was found in a subgroup of women receiving naturopathic medicine (86% of the naturopathic patients), compared with orthopedic care.^33^
- Also in Germany, a poster abstract reported a prospective clinical trial in chronic musculoskeletal pain (including back pain), conducted over 2 weeks of inpatient treatment (*n* = 221). Naturopathic care decreased mean pain scores by 15.1 from 60.7 ± 23.0 (admission) to 45.6 ± 26.2 (1 year post-treatment) (*p* < 0.0001).^14^
- In Canada, a RCT conducted in rotator cuff tendinitis (*n* = 85) compared a set naturopathic treatment protocol to a standardized exercise routine/placebo supplementation over 12 weeks. Naturopathic treatment decreased the Shoulder Pain and Disability Index by 54.5% (*p* < 0.0001) compared to 18% (*p* = 0.0241) in the control group.^27^
- In the US, a multiple sclerosis RCT (*n* = 45) compared naturopathic treatment with “usual care” and with “usual care plus education”. They found no significant differences between groups on any outcome measure at 6 months.^19^
- Another US RCT investigated naturopathic treatment in comparison to Traditional Chinese Medicine (TCM) and specialized dental care (*n* = 160) for TMD. For worst pain, the improvement in the naturopathic medicine group was statistically significant compared to specialized dental care (−1.02 ± 0.45, *p* = 0.025). Naturopathic treatment provided significantly greater decreases than either TCM (*p* < 0.034) or specialized dental care (*p* < 0.012) in TMD-related psychosocial interference.^17^
- Also in the US, subjects with pain from any cause as a feature of presentation were randomized to acupuncture, chiropractic, or naturopathic medicine treatment (*n* = 94). Naturopathic treatment significantly reduced pain from baseline to end of treatment (*p* < 0.0001), but was not statistically significantly different from the other groups.^18^

Overall, this diverse group of studies shows that naturopathic treatment decreased pain scores to a degree comparable or better than standard care or other active treatment controls.

#### Mood disorders

Four studies reported on naturopathic treatment for mood disorders: one in anxiety, one in depression, one on both anxiety and depression, and another on bipolar disorder.

- A Canadian RCT randomized patients to standardized psychotherapy with or without naturopathic care for moderate-to-severe anxiety (*n* = 75).^28^ Beck Anxiety Inventory scores decreased by 56.5% (*p* < 0.0001) in the naturopathic treatment group compared to 30.5% (*p* < 0.0001) in the psychotherapy-only group.
- A US prospective cohort study (*n* = 60) showed significant improvements using naturopathic treatment, with symptomatic improvement in depression (16.4 vs. 8.6, *p* < 0.0001) and anxiety (12.4 vs. 7.2, *p* < 0.0001) scores in patients who returned for two or more visits.^15^
- A small Australian observational study^42^ (*n* = 8) showed improved scores on all areas of the Depression Anxiety Stress Scale-21 (DASS-21) and significant improvement in clinical outcomes with naturopathic treatment in patients who returned for two or more visits (*p* < 0.005).
- In the US, a retrospective case series reported on a small subset of patients diagnosed with treatment-resistant bipolar disorder (*n* = 7) receiving >1 year of naturopathic treatment.^16^ Results were varied, but showed mood stabilization along with withdrawal of psychotropic medication.

Overall, significant reductions in anxiety and depression levels were shown across this group of studies.

#### Complex chronic disease

A total of five studies were included for complex chronic disease, comprising a broad group of mixed chronic conditions within individuals. The primary outcomes included changes in QOL and symptom scores.

- In Germany, three prospective cohort studies examined effectiveness of naturopathic treatment outcomes over a broad group of mixed chronic conditions, in terms of QOL outcomes.^32,34,35^ The studies all used different outcome measures to evaluate treatment effectiveness, with all showing positive results for improving QOL. In one study, the intensity of the main complaint decreased from 59 ± 25 by an average of 25 points at discharge (reduced to 19 points at 6-month follow-up) corresponding to an effect size of 0.86 (0.62 at 6-month follow-up).^32^ The other two studies report statistically significant increases across the majority of QOL domains as measured by two independent QOL scales (*p* < 0.01).^34,35^
- Also in Germany, a cluster-randomized trial (*n* = 58) of naturopathic care compared with usual care in aged residential community living^31^ showed a small-to-medium effect size on a range of geriatric QOL scales.
- In the United Kingdom (UK), a retrospective evaluation of clinic outpatients (*n* = 49) receiving naturopathic care over a 3-year period^45^ showed improvement (*p* < 0.001) between the first and last consultation in patients' symptom scores.

While difficult to group together, this varied group of studies shows an overall positive effect on QOL and symptomatic improvement with naturopathic care.

#### Asthma

Two studies of naturopathic treatment for asthma were included.^39,40^ Both studies were conducted in India and both showed that naturopathic inpatient treatment improved clinical asthma profiles.

- A nonrandomized crossover trial (*n* = 37)^40^ examined 21 days of standard drug therapy at home followed by, and compared to, 21 days of intensive naturopathic inpatient treatment. Results showed significant increase across all measured lung function parameters (*p* < 0.001) and reduced eosinophil counts after naturopathic treatment (*p* < 0.01).^40^
- A retrospective evaluation of patients (*n* = 134)^39^ also investigated intensive naturopathic inpatient treatment for 21 days and showed a significant increase in some indices of lung function at all time points, from postadmission to 6 months (*p* < 0.0035),^39^ with increased peak expiratory flow rate maintained to a 36-month follow-up.

Overall, these two Indian studies show significant positive results for intensive inpatient naturopathic treatment of asthma in lung function parameters.

#### Mixed conditions

This group contains conditions where only a single study was found. The areas of these conditions are as follows: cancer,^46^ menopause,^25^ hepatitis C,^24^ multiple sclerosis,^19^ polycystic ovary syndrome (PCOS),^44^ and HIV (as an adjuvant to antiretroviral therapy).^41^

- In liver cancer, a single retrospective analysis of naturopathic treatment in Japan (*n* = 101) showed a dramatically improved survival rate when *Cordyceps sinensis* was a component of the multi-modality treatment administered.^46^
- In a retrospective cohort study on menopause in the US (*n* = 79), researchers compared naturopathic treatment with usual care (*n* = 160). The most significant improvements resulting from naturopathic treatment occurred for insomnia and decreased energy, with a sevenfold improvement over usual care. Improvements in other symptoms were comparable with improvements in the control group.^25^
- In a retrospective case series of patients with Hepatitis C undertaking naturopathic treatment (*n* = 14), all participants showed reductions in serum alanine aminotransferase (ALT) (average 35 U/L). Seven cases showed an ALT reduction of more than 25%.^24^
- An RCT in multiple sclerosis found no significant differences between usual care, usual care plus education, and usual care plus naturopathic treatment.^19^ However, statistical trends favoring the naturopathic treatment group were found in a single subscale of SF-36 (general health) and in the timed-walk and symptoms of neurologic impairment (EDSS).
- An RCT in overweight PCOS patients (*n* = 122) found highly significant improvement (*p* < 0.001) in the primary outcome of oligomenorrhea/amenorrhea after 3 months of treatment.^44^ The trial compared effect of a LI alone, with LI plus a botanical medicine protocol. Menstrual cycle length was 43 days lower for women in the herbal medicine group (95% CI: 21–65, *p* < 0.001) than for those in the lifestyle-only group, with a large effect size. Improvements across other areas, including BMI, insulin, hormone levels, stress, and pregnancy, also were seen.
- A prospective, parallel, matched-control study of naturopathic and yoga interventions as adjuvant treatments to antiretroviral therapy in a group with HIV, conducted over 1 month, showed improved CD4 count in the intervention group over the control group which received only antiretroviral therapy (*p* = 0.047).^41^

##### Heterogeneity

Clinical heterogeneity (defined as differences in participants, treatments, outcome characteristics, or research setting)^47^ in this scoping review is substantial. While all the interventions are similar in intervention type (whole-system, multi-modality naturopathic medicine), they vary substantively at the patient level (condition, baseline severity, age, gender, ethnicity, and comorbidities); intervention level (duration and comparator/controls); outcome level (outcome measure and definitions); and in research setting. The authors did not test for statistical heterogeneity.

##### Study quality and risk of bias

There is a wide range of quality in the included studies, given the breadth of the research reported. Cochrane risk of bias assessments were completed on the nine RCTs (Fig. 3), showing low risk of bias for all areas, except blinding of participants and personnel, and moderate bias for allocation concealment. In observational research, selection bias is considered high for several studies in which retrospective data have been reported and where outcomes include only patients who have returned for multiple visits. Reporting bias also is likely to be high in retrospective studies in which isolated outcomes are reported. Several of the prospective cohort studies are well-conducted, with a low level of bias, particularly those, such as Braun et al.,^43^ Teut et al.,^31^ and Bradley et al.,^22^ that compared results with a “usual care” cohort.

## Discussion

This systematic scoping review identified a diverse collection of quantitative whole-system, multi-modality naturopathic medicine research from around the world reported in 33 publications. A majority of the research (15 articles) was conducted in North America, where the modern naturopathic medical profession has developed^48^ and where the first school of naturopathic medicine was founded by Benedict Lust in 1902.^1^ Other countries contributing research include Germany (six studies), where naturopathy is rooted in the development of hydrotherapy by founders Priessnitz and Kneipp,^49^ and India (six articles) where naturopathy was popularized and influenced by MK Gandhi, regarded as the Father of the Indian nation, at the turn of the 20th century.^50^

### Clinical outcomes

Although results from these studies are highly diverse, they also are predominantly positive, showing improved health outcomes and QOL across conditions and across nationalities. These studies demonstrate a broad range of naturopathic modalities, against a background of different practitioner training, legislative and regulatory jurisdictions, and different research approaches. Their results concur with Oberg et al.^9^ who determined the effect sizes of the primary medical outcomes for 13 North American studies, concluding that there were positive outcomes and improved QOL in individuals with, or at risk for, chronic conditions, including CVD,^23,29^ type 2 diabetes,^20–22^ chronic pain,^17,18,26,27^ anxiety,^28^ hepatitis C,^24^ and menopausal symptoms.^25^ The authors have updated and expanded this review to the global literature, thereby increasing the range of positive outcomes to include depression and anxiety,^15^ bipolar disorder,^16^ asthma,^39,40^ PCOS,^44^ and increased cancer survival time.^46^ It also adds additional studies to support positive outcomes for CVD,^13,37,38,43^ type 2 diabetes,^36^ chronic pain,^14,32^ and anxiety and mood disorders.^42^

Three of the German studies examined mixed chronic conditions and demonstrated positive outcomes for QOL and perceived pain in: older adults (mean age 79.4 years) living in nursing homes^31^ and older adults (mean age 57.3 years) admitted to hospital for allergic complaints and rheumatic, chronic-bronchial, and metabolic diseases.^34,35^ In addition, one UK study of mixed chronic conditions demonstrated positive outcome for overall symptom improvement.^45^

The benefit of naturopathic treatment as an adjunct to antiretroviral treatment in individuals with HIV could not be assessed, because the study^41^ lacked the data required to make a clinical assessment of the effect.

The study on multiple sclerosis^19^ showed no difference in the primary outcome (the QOL short form 36; SF-36) between the three intervention groups (usual care, usual care plus naturopathic care, or usual care plus education). Shinto et al.^19^ concluded that positive outcome trends in individuals with multiple sclerosis warranted further evaluation.

### Community versus inpatient studies

A main characteristic of the North American research is that all the studies were undertaken in free-living individuals treated in a community setting. By comparison, all six of the Indian studies, a single UK study, and five of the six German studies were undertaken in inpatients admitted to a treatment facility. The three Australian studies were mixed, with two in community practice and one in an inpatient setting (Table 9).

In Germany, naturopaths (*heilpraktikers*) are licensed by the state and comprise 40,000 of the 60,000 naturopathic professionals in Europe.^51^ Heilpraktikers are nonmedical practitioners trained in the philosophy and modalities of naturopathic medicine and trace their roots to the origin of the profession. To date, no research into whole-system naturopathic medicine by Heilpraktikers could be located with an English title and abstract. In addition to Heilpraktikers, there is a group of medical doctors (*naturheilkunde)* who specialize in naturopathic modalities and self-identify as providing naturopathic treatment.^49^ All studies from Germany included in this review were undertaken by these naturopathic practitioners in both inpatient and in residential facilities.

A meta-analysis^52^ of eight studies on the effect of German inpatient integrative medicine research on QOL included four of the studies included in this scoping review. A random effect meta-analysis of the eight studies revealed an overall effect size of 0.37 (95% CI: 0.28–0.45) in the physical score and 0.38 (95% CI: 0.30–0.45) in the mental score of the SF-36, demonstrating the effectiveness of the inpatient treatment model used in Germany.

### Different countries, different designs, similar conclusions

An insight into the diversity of naturopathic treatment, and the outcomes that can be achieved, can be gained by contrasting two different studies on chronic lower back pain. One study was undertaken in Canada^26^ in an outpatient setting in individuals with chronic lower back and the other in Germany^33^ in a cohort who required hospitalization for chronic lower back pain.

The Canadian naturopathic treatment consisted of acupuncture, relaxation techniques, and dietary recommendations (diet high in omega three fatty acids, magnesium, and calcium). In Germany, classical naturopathic treatment is codified for use in acute inpatient settings with a minimum requirement that five of these eight therapies are applied: (1) nutrition therapy; (2) hydrotherapy/thermotherapy; (3) other physical modalities; (4) phytotherapy; (5) lifestyle-regulatory therapy; (6) exercise therapy; (7) detoxification procedures; or (8) an additional procedure (manual therapy, acupuncture/Chinese medicine, homeopathy, neural therapy, or art/music therapy).^53^

Both studies used the Oswestry Disability Index as the primary outcome measure and demonstrated that whole-system multi-modality naturopathic medicine made a significant difference in comparison to controls. The Canadian study demonstrated that naturopathic treatment is more effective than education and exercise for chronic lower back pain. The German study demonstrated that it is comparable with mainstream orthopedic treatment when back pain is so acute as to warrant inpatient care and that it is potentially better than mainstream orthopedic care for women. Together, these two very different studies demonstrate a stronger case for the effectiveness of whole-system multi-modality naturopathic treatment of chronic lower pain.

### Regional differences and generalizability

The WNF has determined that there is a high degree of global consistency in the core concepts that define naturopathic medicine and that all countries utilize a common set of naturopathic modalities.^54,55^ The consistent positive outcomes in similar conditions in different countries are likely to reflect this commonality.

Regional differences, however, may affect the generalizability of studies, if the scope of practice used in a specific study includes treatment modalities that are not accessible or utilized in other regional areas for historical, legal, or educational reasons.

Specific regional differences exist in naturopathic practice concerning modalities emphasized (e.g., the addition of acupuncture in Canada or pharmaceutic prescribing rights in some areas of the US). In some jurisdictions, there are core modalities underlying naturopathic practice, such as yoga in India or the combination of osteopathic techniques with naturopathic practice in the UK. Thus, the UK study on the treatment of musculoskeletal conditions using a mixture of osteopathic and naturopathic techniques^45^ is consistent with naturopathic practice in that country.

The diverse practice settings and the extensive range of modalities represented in the research provide a sound argument for expanding the scope of practice in jurisdictions where generalizability is limited. The extent to which the studies included in this scoping review are generalizable to other countries must be assessed on a study-by-study basis.

### Pragmatic trials versus therapeutic tools

Pragmatic whole-system (whole-practice) research provides a “real life” snapshot of how naturopathic medicine is practiced in the community, reflecting the naturopathic individualized approach to treatment and ongoing management. In the absence of whole-system data, the only way to effectively and objectively evaluate a discipline is to assess its major therapeutic tools. In naturopathic medicine this would include evidence for dietary and lifestyle interventions and specific botanical medicines and nutritional supplements.

In 2005, a review was undertaken of naturopathy and of Western herbal medicine in Australia.^5^ The report concluded that while evidence for the whole-system practice of naturopathic medicine was lacking, a range of nutritional supplements and botanical medicines (the “tools of trade”) demonstrated benefits at the highest levels of evidence and have proven efficacy.

In conventional medicine, evidence for the effectiveness of their “tools of trade” (pharmaceutics and surgery) is generally considered sufficient to demonstrate the effectiveness of its practice. By comparison, a recent governmental review of naturopathic medicine in Australia judged the practice of naturopathic medicine solely on the scope of whole-system research, limited to only systematic reviews containing RCTs published since 2008.^11^ Based on this limited scope, they concluded that naturopathic medicine's overall effectiveness could not be proven, and the Government has proposed exclusion from private health insurance effective from April 1st, 2019.

To provide a more comprehensive method for assessing the effectiveness of naturopathic medicine, there is a real need for a new type of effectiveness review. Such a review would systematically evaluate evidence for specific therapeutic agents used by naturopathic clinicians combined with the results of pragmatic clinical trials on whole-system naturopathic practice, in a specific condition or population. It is not sufficient, nor appropriate, to rely on either aspect alone as the sole method of assessment of the effectiveness of naturopathic medicine.

### Context of EBM

The EBM movement began in conventional medicine due to a concern that clinical decision-making was not evidence based.^56^ The scope of conventional medicine is so large that charting the extent of its total evidence at any given point in time is problematic. In 2007, *BMJ Clinical Evidence* reviewed 2500 treatments supported by good evidence. It rated 15% of treatments as beneficial, 22% as likely to be beneficial, 7% as partly beneficial and partly harmful, 5% unlikely to be beneficial, and 4% likely to be ineffective or harmful. For the remaining 47%, the effect of treatment was rated as currently not demonstrated.^57^ If this review was undertaken today, these numbers would differ; however, it is important to recognize from these figures that conventional medicine, like all fields in health care, has extensive work to do regarding the evidence on which practice is based.

In contrast to classical RCTs which have dominated EBM and which utilize a reductionist approach that fails to recognize the complexities of real-world clinical practice,^2^ the research outlined in this scoping review is pragmatic in nature and sets out to determine the effectiveness of a whole-system approach in real-world clinical practice.

### Limitations

There are several limitations inherent in grouping such as a broad range of heterogeneous studies. No specific analysis of the effectiveness of naturopathic treatment was conducted, due to the breadth of study types, outcomes assessed, treatment settings, and modalities used. The most robust studies reported results in comparison with usual care; however, comparative controls were used in only 4 of 12 prospective studies^18,22,33,43^ and in one of eight retrospective studies.^25^ In addition, there is a high risk of reporting and selection bias in many of the observational studies (i.e., criteria such as multiple return visits to naturopathic centers for patient inclusion in the dataset).

The types of modalities used and the intensity of treatments are highly variable across the studies reported here. It is not possible to compare outcomes for intensive inpatient treatment with several visits spaced over 6 months in a community setting. As such, the aim of this scoping report was not to compare the effectiveness of the research, but to show the breadth of the research into whole-system naturopathic medicine.

There is little distinction between some included studies where “integrative medicine” is applied and some of the excluded studies using integrative medicine. The acceptance criterion was that treatments were administered by a self-identified naturopath, as opposed to a conventional doctor or nurse; however, the authors recognize that some studies of integrated hospital care might not represent naturopathic medicine as clearly as studies that have naturopathic-only treatment. Also some studies of treatment provided by naturopaths might have been overlooked if this was not specified in the text. In addition, inclusion criteria for non-English language articles might have precluded relevant studies.

The decision to limit inclusion of case series to those with five or more cases was arbitrary and might have excluded some studies. Although seven was the median number of cases included in articles with titles specifying “case series,” the number of cases included is not a differentiating characteristic.^58^ The decision was taken to ensure that any inference about clinical practice was based on multiple observations.

The majority of studies assessed were positive, which carries a specific concern regarding publication bias (the possibility that negative studies might have been undertaken, but not reported). This is known to be true in pharmaceutic research,^59^ but currently difficult to assess in naturopathic medicine.

## Conclusions and Future Directions

The global naturopathic research landscape contains a small, but expanding body of practice-based, whole-system, multi-modality research. To date, research with higher methodological quality shows that whole-system multi-modality naturopathic medicine is effective for treating a range of conditions, including cardiovascular disorders, musculoskeletal pain, type 2 diabetes, PCOS, depression, and anxiety. Research with lower methodological quality also suggests that naturopathic medicine is effective for treating chronic pain, hepatitis C, menopausal symptoms, bipolar disorder, and asthma and in increasing cancer survival time. Results were positive across world regions for similar conditions, which are likely to reflect the global consistency in applying the core concepts of naturopathic practice utilizing the common set of naturopathic modalities.

Although there is a vast array of clinical trial evidence supporting the tools of trade used in naturopathic medicine (dietary and lifestyle interventions and specific botanical medicines and nutritional supplements), there is a distinct lack of well-conducted pragmatic trials evaluating the complex intervention of whole-system, multi-modality naturopathic care. Until substantively more whole-system research is undertaken, evaluating the effectiveness of naturopathic medicine requires a combination of both these types of evidence.

There is a need for pragmatic, real-world trials in which complex naturopathic treatment is compared with usual care to build a high-quality evidence base on the effectiveness of whole-system, multi-modality naturopathic practice. This need has recently led to development of a research consortium of naturopathic academic clinics in four countries and across multiple world regions to develop robust, international, multicenter collaboration.^60^ This consortium has been endorsed by the World Naturopathic Federation, with the goal to significantly increase the amount and quality of global naturopathic whole-system research.
